# Cyberbullying victimization and suicidal ideation among in-school adolescents in three countries: implications for prevention and intervention

**DOI:** 10.1186/s12888-023-05268-9

**Published:** 2023-12-14

**Authors:** Prince Peprah, Michael Safo Oduro, Reforce Okwei, Collins Adu, Bernard Yeboah Asiamah-Asare, Williams Agyemang-Duah

**Affiliations:** 1 Social Policy Research Centre, University of New South Wales, Sydney, Australia; 2 Centre for Primary Health Care and Equity, University New South Wales, Sydney, Australia; 3 Department of Applied Statistics and Research Methods, University of Northern Colorado, Greeley, CO USA; 4 Pfizer Worldwide Research and Development, Pharm Sci and PGS Statistics, Groton, CT USA; 5 Department of Geography and Environment, Western University, 1151 Richmond Street, N6A 3K7 London, Ontario Canada; 6 Department of Health Promotion, Education and Disability Studies, Kwame Nkrumah University of Science and Technology, Kumasi, Ghana; 7 Centre for Social Research in Health, University of New South Wales, Sydney, NSW Australia; 8 Curtin School of Population Health, Curtin University, Kent Street, Bentley, WA Australia; 9 Health Psychology, Institute of Applied Health Sciences, University of Aberdeen, Aberdeen, UK; 10 Department of Geography and Planning, Queen’s University, ON K7L 3N6, Kingston, Canada

**Keywords:** cyberbullying victimization, Suicidal ideation, Risk factors, Protective factors, Adolescents, Argentina, Panama, St Vincent and the Grenadines

## Abstract

**Background:**

Countries in South and Central America and the Caribbean are among the countries with the highest adolescent cyberbullying crimes. However, empirical evidence about the effect of cyberbullying victimization on suicidal ideation among in-school adolescents in these countries remains limited. The present study examined the association between cyberbullying victimization and suicidal ideation among in-school adolescents in Argentina, Panama, St Vincent, and the Grenadines.

**Methods:**

A representative cross-sectional data from 51,405 in-school adolescents was used. Hierarchical logistic regression analysis was used to estimate the association between cyberbulling victimization and suicidal ideation.

**Results:**

Overall, 20% and 21.1% of the adolescents reported cyberbullying victimization and suicidal ideation, respectively in the past year before the survey. Suicidal ideation was higher among adolescents who experienced cyberbullying victimization (38.4%) than those who did not experience cyberbullying victimization (16.6%). Significantly higher odds of suicidal ideation were found among adolescents who had experienced cyberbullying victimization than those who had not experienced cyberbullying victimization [aOR = 1.88, 95% CI: 1.77–1.98].

**Conclusion:**

This finding calls for developing and implementing evidence-based programs and practices by school authorities and other relevant stakeholders to reduce cyberbullying victimization among adolescents in this digital age. Protective factors such as parental support and peer support should be encouraged.

## Background

The advancement of technology and digital innovations like the internet have changed how individuals interact [1]. Though these changes have permeated several facets of life and allowed for significant developments, they have also resulted in transgression and oppression and have made many forms of abuse more pervasive [1]. Proof of the abuse of the internet, like online web portals and social media applications (Facebook, WhatsApp, Emails, Instagram, YouTube), relates to how traditional bullying has transformed into cyberbullying victimization and expanded the scope beyond physical settings [2]. Cyberbullying encompasses sending harrying or intimidating messages and mailing embarrassing statements online [3] against people who cannot defend themselves easily [4]. Cyberbullying acts are indirect rather than face-to-face so that cyberbullies can stay unidentified and unseen. A single cyberbullying action can escalate swiftly and be replicated by a wider audience than repeated traditional bullying acts [5]. Additionally, the nature and form in which cyberbullying victimization occurs make it ideal for perpetrators to escalate the extent, frequency, the severity of bullying and retraumatizing victims in diverse ways [6].

Although cyberbullying victimization occurrence differs across countries and continents due to various definitions of cyberbullying victimization [2], researchers like Slonje and Smith [7] have argued on the back of changing technology forms that rates of cyberbullying victimization were on the ascendency. In a survey involving 1,301 adolescents between the ages of 13 and 17 across 13 states in the United States of America, 54% of respondents reported having been cyberbullied on social media platforms (Facebook, YouTube, Twitter, and Instagram) [8]. Mishna et al. [9], after examining 2,186 self-reported data of middle and high school students in Canada, found that over 50% of the students identified themselves as victims or culprits of cyberbullying. Cyberbullying victimization among adolescents continues to concern many countries and international bodies. A recent report by United Nations Children’s Fund (UNICEF) indentified one-third of over 170,000 adolescents and young adults in 30 countries as victims of cyberbullying [10].

The experiences and consequences of cyberbullying, especially among adolescents, are more brutal, devastating, and multiple because of the extensive reach of the internet to several people. The texts and materials can be stored online, causing the victims of cyberbullying to relive demeaning experiences, often including suicidal behaviors such as suicidal ideation and attempt [3, 11]. Suicidal behaviors relate to actions done by people to die and comprise suicidal ideation, a suicide strategy, suicide efforts, and death by suicide [12].

Suicidal behaviors among adolescents globally have become a serious concern, with 3,000 suicide deaths among youth aged between 10 and 19 years in 2018 in the United States (US) alone [13]. Again, data from the 2017 Youth Risk Behavior Survey (YRBS) in the US indicate that 17.2% of adolescents seriously contemplated suicide, 13.6% made plans to attempt suicide, and 7.4% attempted suicide [14]. Elia et al. [15] analyzed the Global School-based Health Survey (GSHS) of adolescents aged 10–19 from 21 Latin American and Caribbean Countries and found that 7.5% of boys and 17.5% of girls reported suicidal thoughts with planning over the last year. However, their study was limited to the association between overweight/obesity and suicidal ideation. The study did not focus on cyberbullying victimization and included only one of the countries (Argentina) studied in the present study.

Suicidal behavior among adolescents is induced by various factors, which include individual factors (social media, age, gender, depression), family factors (parental marital status, household income, culture of maltreatment), cyberbullying victimization, and socio-environmental factors (type of school, social support from parents and peers) [16]. Research examining cyberbullying victimization and suicide behaviors such as suicide ideation has been growing in recent years [17]. Several studies have indicated that cyberbullying victimization is strongly related to suicidal behaviors among adolescents [18], and victims of cyberbullying victimization have double the risk of suicidal behavior than non-victims [10]. Another study among 1,963 students in 30 middle schools in the US has also found cyberbullying victims to be 1.9 times more likely to attempt suicide [19].

Despite the growing literature on the association of cyberbullying victimization with suicidal behaviors research, limited evidence exists among Latin American and Caribbean countries, particularly Argentina, Panama, St Vincent and the Grenadines. However, the region has copious reports of cyberbullying victimization incidents [20]. For instance, findings from the Global Kids Online survey by UNICEF in 2017 indicated that 13–17 percent of internet users report negative experiences, including cyberbullying victimization in internet use, was 77% in Argentina [21]. Other News reports also showed that adolescent cyberbullying victimization increased by 50% between 2020 and 2021, with 7 out of 10 children and adolescents reporting cyberbullying victimization [22]. Evidence again found that cyberbullying in Panama, St Vincent, and the Grenadines is rising [23]. Despite the prevalence of cyberbullying victimization in these countries, much remains unexplored, mainly its association with suicidal ideation.

In this study, we examined data from the Global School-Based Health Survey (GSHS) in Argentina, Panama, St Vincent, and the Grenadines to measure the association between cyberbullying victimization and suicidal ideation among adolescents between the ages of 11 and 17 who were in school. Based on past studies [24, 25], we conceptualize cyberbullying victimization as a stressful experience and thus hypothesize that victims of cyberbullying will be more likely to report more significant suicidal ideation.

## Methods

### Data and participants

This study used data from the Global School-based Student Health Survey (GSHS) among three countries. The GSHS is a large and representative survey of students’ health behaviors and risk factors. The survey was developed by the World Health Organization (WHO) and the US Centers for Disease Control and Prevention (CDC), and other United Nations (UN) allies and country-specific institutions [26]. The survey draws content from the CDC Youth Risk Behaviour Survey (YRBS), for which test-retest reliability has been established [27]. Information, including the aims, methodology, and sampling procedure of the GSHS, is available at https://www.cdc.gov/.

Briefly, the participants for the GSHS are primarily school-going adolescents aged 13–17 years. The sampling procedure included a standardized two-stage probability sampling approach for the participant selection procedure within each participating country. In the first stage, the schools were selected through probability proportional to size sampling design. The second stage involved randomly selecting classrooms, primarily including students aged 13–15 years in each selected school. Irrespective of age, all students in the selected classrooms were eligible to participate in the survey. This study used the most recent GSHS data available in Argentina, Panama, St Vincent, and the Grenadines. This selection ensured that the analysis reflects and represents recent or current trends. Data were weighted for non-response and probability sampling to enable the generalization of results to the eligible population. The complete data on all variables included in the analysis for this study was available for 51,405 adolescents. Hence, this constitutes the analytical sample in this study. The characteristics of each country, including the country name, survey year, and sample size, are provided in Table 1.

**Table 1:** Distribution of study sample across countries

Country name	Year of survey	Weighted N	Weighted %
Argentina	2018	47,545	92.5
Panama	2018	2392	4.7
St Vincent and the Grenadines	2018	1468	2.8
**Total**		**51,405**	**100**

### Data collection procedure

Data collection was conducted during a regular class period with the help of trained enumerators from local social work organizations and other research institutions. The GSHS questionnaire was close-ended, structured, and developed in English but translated into the local language in each country. The questionnaire included multiple-choice questions. The questionnaires were administered after informed consent was obtained from the students, parents, and school officials. The trained enumerators explained the aim of the study and the instructions in detail for the students before the questionnaires were distributed. In addition, the survey ensured that students’ privacy was protected through anonymous and voluntary participation. The questionnaires were answered, and participants recorded their responses on computer scannable sheets. The study complied with the Declaration of Helsinki, and each country’s Institutional review boards or ethics committees reviewed and approved all GSHS surveys. Also, details of the systematic procedures used for the data collection among the students can be found at https://www.cdc.gov/.

### Measures

#### Cyberbullying victimization (exposure variable)

The exposure variable in this study was cyberbullying victimization. Experience of cyberbullying victimization in the past 12 months before the survey was assessed by a single item: *“During the past 12 months, have you ever been cyberbullied?”* Cyberbullying victimization includes students being bullied through texting, Instagram, Snapchat, Facebook, WhatsApp, or other social media. The response was binary, yes, coded as “1,” and no, coded as “0”. Studies conducted among adolescents in Hong Kong [28], Vietnam [16], and Argentina [29] used the exact measurement.

#### Suicidal ideation (outcome variable)

The outcome variable investigated in this study was suicidal ideation. This variable was measured based on a response to the question: *“During the past 12 months, did you ever seriously consider attempting suicide?”* The responses to this question were “yes” and “no.” Students who responded yes were coded as “1,” and those who answered no were coded as “0”. Previous studies investigating suicide ideation among adolescents have used the exact measurement [16, 24, 28, 29].

#### Explanatory variables

Participants self-reported all demographics and health-related variables; these were selected based on previous evidence of their associations with cyberbullying victimization and suicidal ideation [16, 24, 28, 29] and included in regression models as control variables. We had variables such as age (years), gender, alcohol intake, cigarette smoking, food insecurity, tobacco use, drug use, physical activity, parental involvement, physical violence, loneliness, friendship, and peer support. Details on the measures and variables coding are provided in Table 2.

### Statistical methods

Data from the 2018 Global School-based Student Health Survey conducted in the three countries (Panama, St. Vincent and the Grenadines, and Argentina) are aggregated and analyzed via descriptive and inferential statistical methods. The illustrative process involves summary statistics related to demographic data and other key independent health-related variables collected on survey respondents. This is followed by two inferential statistical methods: Pearson’s Chi-square test of independence [30] and the Binary Logistic Regression Model [31]. More broadly, the Chi-Square test of independence is employed to assess the bivariate association between the response variable, suicidal ideation, and every other independent categorical variable (inclusive of these are gender, cyberbullying, loneliness, alcohol intake, cigarette smoking, tobacco use, drug use, physical activity, parental support, food insecurity, peer support, and friendship). The null hypothesis of no relationship between suicidal ideation and the selected independent study variables is tested. If the resulting p-values are less than a statistical level of significance $(\alpha =0.05)$, the null hypothesis is rejected. This will imply strong evidence of a significant bivariate relationship between suicidal ideation and the independent categorical variables. Afterward, a Binary Logistic Regression Model is fitted to the data. This is performed hierarchically. First, a simple binary logistic model involving one predictor (cyberbullying victimization) is fitted to the data, and model evaluation is performed. A multivariable binary logistic regression, including the cyberbullying predictor and all other covariates selected, follows this. Model evaluation is performed via the Akaike Information Criterion (AIC) [32], which compares a set of statistical models with each other and determines which of them is a better fit for the given data. Smaller values of the AIC measure indicate a better fit of the model. Based on the model selected, statistical inference is predicated on odds ratios of estimated parameters, 95% confidence intervals of the estimated odds ratios, and p-values. Inference is made at a 5% significance level, and all statistical analyses are performed in R software version 4.2.0.

## Results

### Participants’ characteristics and prevalence of cyberbullying and suicidal ideation

Table 2 describes the background characteristics, health risks, health-promoting variables, and the prevalence of cyberbullying victimization and suicidal ideation among the participants. In all, 51,405 adolescents were included in the study. These participants involved 27,226 (53%) males and 24,179(47%) females. In terms of health risk behaviors, over 40% felt lonely(43.5%), more than half consumed alcohol(54.6%), and a small proportion smoked cigarettes(17.7%) and used drugs(14.9%). On the other hand, most of the participants engaged in physical activity (83.2%), received parental support (59.4%), received peer support (72.6%), and had one or more friends (94.3%). The prevalence of cyberbullying victimization experience was 20.4%, and suicidal ideation was 21.1% among adolescents from the three countries (see Table 2).

**Table 2:** Descriptive statistics of study variables of All 3 countries (Panama, Argentina, and St Vincent and Grenadines)

Variables	Levels/Categories	Sample	Sample Percentage
**Main Outcomes**			
Suicidal Ideation	Yes	10,830	21.1
	No	40,575	78.9
Cyberbullying victimization	Yes	10,495	20.4
	No	40,910	79.6
**Demographic Factors**			
Gender	Female	24,179	47
	Male	27,226	53
Age (years)	11 to 13 years old or younger	9392	18.3
	14 years old	11,494	22.4
	15 years old	11,530	22.4
	16 years old	10,731	20.9
	17 years old	7622	14.8
	18 years old or older	636	1.2
**Protective Factors**			
Peer Support	Never	4049	7.9
	Rarely	10,005	19.5
	Sometimes	13,425	26.1
	Most Times	14,825	28.8
	Always	9101	17.7
Friendship (Number of friends)	0	2899	5.6
	1	4366	8.5
	2	8340	16.2
	3 or more	35,800	69.6
Physical activity	No	8612	16.8
	Yes	42,793	83.2
Parental Support	Never	11,222	21.8
	Rarely	9709	18.9
	Sometimes	9078	17.7
	Most Times	9024	17.6
	Always	12,372	24.1
**Risk Factors**			
Loneliness	Never	15,905	30.9
	Rarely	13,138	25.6
	Sometimes	13,766	26.8
	Most of the Time	5810	11.3
	Always	2786	5.4
Alcohol Intake	No	23,327	45.4
	Yes	28,078	54.6
Cigarette smoking	No	42,282	82.3
	Yes	9123	17.7
Tobacco use	No	47,941	93.3
	Yes	3464	6.7
Drug use	No	43,768	85.1
	Yes	7637	14.9
Food Insecurity	Never	34,543	67.2
	Rarely	10,840	21.1
	Sometimes	5070	9.9
	Most Times	688	1.3
	Always	264	0.5

### Distribution of suicidal ideation across cyberbullying victimization and explanatory variables

Table 3 shows the distribution of suicidal ideation across cyberbullying victimization and covariates. The results showed significant differences in suicidal ideation across cyberbullying victimization among the participants (p < 0.0001). Specifically, suicidal ideation was higher among adolescents who experienced cyberbullying victimization (38.4%) than those who did not experience cyberbullying victimization (16.6%). All the explanatory variables were significantly related with suicidal ideation (p < 0.0001).

**Table 3:** Chi-Square test of independence for the association between suicidal ideation and study covariates

Variables	Levels/Categories	Suicidal Ideation		Chi-Square Test Statistic, P-value
		Yes	No	
**Main Outcomes**				
Cyberbullying victimization	Yes	38.46% (4036)	61.54%(6459)	2396.4, < 0.0001
	No	16.61% (6794)	83.39% (34,116)	
**Demographic Variables**				
Gender	Female	13.25%(3203)	86.75% (20,976)	1678.3, < 0.0001
	Male	28.01%(7627)	71.99%(19,599)	
Age (years)	11 years old or younger	19.30% (1813)	80.70% (7579)	38.038, < 0.0001
	14 years old	21.15% (2431)	78.85% (9063)	
	15 years old	22.72% (2620)	77.28% (8910)	
	16 years old	21.01% (2255)	78.99%( 8476)	
	17 years old	20.83% (1588)	79.17%(6034)	
	18 years old or older	19.34%(123)	80.66%(513)	
**Protective Variables**				
Peer Support	Never	29.32% (1187)	70.68% (2862)	821.69, < 0.0001
	Rarely	28.35% (2836)	71.65%(7169)	
	Sometimes	21.47% (2883)	78.53% (10,542)	
	Most Times	17.38% (2577)	82.62% (12,248)	
	Always	14.80% (1347)	85.2%(7754)	
Friendship (Number of friends)	0	33.11%(960)	66.89%(1939)	764.82, < 0.0001
	1	29.45% (1286)	70.55%(3080)	
	2	25.90% (2160)	74.1%(6180)	
	3 or more	17.94% (6424)	82.06% (29,376)	
Physical activity	No	24.77% (2133)	75.23%(6479)	84.888, < 0.0001
	Yes	20.32% (8697)	79.68% (34,096)	
Parental Support	Never	35.78% (4015)	64.22% (7207)	2914.9, < 0.0001
	Rarely	27.11% (2632)	72.89% (7077)	
	Sometimes	19.17% (1740)	80.83% (7338)	
	Most Times	13.01% (1174)	86.99% (7850)	
	Always	10.26% (1269)	89.74% (11,103)	
**Risk Factors**				
Loneliness	Never	6.78%(1078)	93.22% (14,827)	9149.3, < 0.0001
	Rarely	11.52% (1513)	88.48% (11,625)	
	Sometimes	25.38% (3494)	74.62% (10,272)	
	Most of the Time	49.93% (2901)	50.07%(2909)	
	Always	66.19% (1844)	33.81%(942)	
Alcohol Intake	No	16.11% (3757)	83.89% (19,570)	631.82, < 0.0001
	Yes	25.19% (7073)	74.81% (21,005)	
Cigarette smoking	No	17.90% (7567)	82.10% (34,715)	1440, < 0.0001
	Yes	35.77% (3263)	64.23%(5860)	
Tobacco use	No	19.81% (9497)	80.19%(38,444)	676.17, < 0.0001
	Yes	38.48% (1333)	61.52%(2131)	
Drug use	No	19.00% (8318)	81.00% (35,450)	753.32, < 0.0001
	Yes	32.89% (2512)	67.11% (5125)	
Food Insecurity	Never	17.34% (5989)	82.66%(28,554)	1226.7, < 0.0001
	Rarely	24.90% (2699)	75.10% (8141)	
	Sometimes	33.91% (1719)	66.09% (3351)	
	Most Times	48.69%(335)	51.31%(353)	
	Always	33.33%(88)	66.67%(176)	

### Logistic regression of the association between cyberbullying victimization and suicidal ideation among the adolescents

Tables 4 and 5 show the regression results regarding the association of cyberbullying victimization with suicidal ideation among adolescents. In all, two models were fitted to examine the associations hierarchically. In model I, only the exposure variable of cyberbullying victimization and the outcome variable of suicidal ideation was considered. The results showed a statistically significant association between cyberbullying victimization and suicidal ideation among adolescents. Specifically, adolescents who experienced cyberbullying victimization were three times more likely to consider attempting suicide compared to those who did not experience cyberbullying victimization [OR = 3.13, 95% CI: 2.99–3.28]. This model had an AIC value of 50,771 (see Table 4).

**Table 4:** Simple binary logistic regression results. model with only cyberbullying victimization variable considered

Variable	OR Estimate	P-value	2.50%	97.50%
(Intercept)	0.199	< 0.0001	0.194	0.204
Cyberbullying victimization (Reference = No)				
Yes	3.137	< 0.0001	2.993	3.289

After adjusting for the various demographic, health, and health-related behaviors variables in Model II, the association of cyberbullying victimization with suicidal ideation was attenuated but remained statistically significant. cyberbullying victimization adolescents were almost two times more likely to consider attempting suicide compared to those who were not cyberbullied [aOR = 1.88, 95% CI: 1.77–1.98] (see Table 5).

In terms of the covariates, adolescent females had a 72% higher odds of having suicidal thoughts compared to boys [aOR = 1.72, 95% CI: 1.63–1.81]. Also, feeling lonely was associated with a higher chance of having suicidal ideation, with the odds increasing as the level of loneliness increased. Smoking, alcohol consumption, drug use, and tobacco use were all associated with a higher odds of having suicidal ideation. Furthermore, being physically active slightly reduced the odds of having suicidal thoughts (about 3% less likelihood of suicidal ideation [aOR = 0.97, 95% CI: 0.91–1.03]. Also, having strong parental support reduced the odds of suicidal thoughts, with higher support levels leading to lower odds. Peer support also reduced the odds of suicidal thoughts, with higher support levels having a similar effect. This model had a relatively lower AIC value of 41,518, indicating a better fit to the data (see Table 5).

**Table 5:** Multiple binary logistic regression: model with all variables considered

Variable	OR Estimate	P-value	2.50%	97.50%
(Intercept)	0.118	< 0.0001	0.102	0.138
Cyberbullying victimization (Reference = No)				
Yes	1.88	< 0.0001	1.780	1.980
Age(reference = 11 years old to 13 years old or younger)				
14 years old	0.945	0.154	0.873	1.020
15 years old	0.85	< 0.0001	0.786	0.919
16 years old	0.695	< 0.0001	0.641	0.754
17 years old	0.643	< 0.0001	0.588	0.703
18 years old or older	0.529	< 0.0001	0.418	0.666
Gender (Reference = Male)				
Female	1.72	< 0.0001	1.630	1.810
Loneliness (Reference = Never)				
Rarely	1.47	< 0.0001	1.350	1.600
Sometimes	3.11	< 0.0001	2.870	3.360
Most Times	7.05	< 0.0001	6.460	7.700
Always	12.3	< 0.0001	11.000	13.700
Alcohol Intake (Reference = No)				
Yes	1.23	< 0.0001	1.170	1.300
Cigarette Smoking (Reference = No)				
Yes	1.57	< 0.0001	1.460	1.680
Tobacco use (Reference = No)				
Yes	1.15	0.006	1.040	1.280
Drug use (Reference = No)				
Yes	1.27	< 0.0001	1.180	1.370
Food Insecurity (Reference = Never)				
Rarely	1.18	< 0.0001	1.110	1.250
Sometimes	1.42	< 0.0001	1.310	1.530
Most Times	2.05	< 0.0001	1.710	2.460
Always	1.32	0.082	0.962	1.800
Physical Activity (Reference = No)				
Yes	0.97	0.344	0.910	1.030
Parental Support (Reference = Never)				
Rarely	0.764	< 0.0001	0.713	0.817
Sometimes	0.572	< 0.0001	0.531	0.615
Most Times	0.445	< 0.0001	0.410	0.483
Always	0.412	< 0.0001	0.381	0.446
Peer Support (Reference = Never)				
Rarely	0.973	0.575	0.884	1.070
Sometimes	0.866	0.003	0.788	0.952
Most Times	0.846	0.001	0.768	0.931
Always	0.872	0.011	0.784	0.969
Friendship (No of Friends) (Reference = 0)				
1	0.958	0.480	0.850	1.080
2	0.858	0.006	0.770	0.957
3 or more	0.766	< 0.0001	0.694	0.846

## Discussion

### Main findings

In this representative multi-country study, the experience of cyberbullying victimization and suicidal ideation was relatively high among adolescents in school. Suicidal ideation was high among adolescents who were cyberbullying victims. After adjusting for several demographic and health lifestyles and health-related factors, the present study results show that cyberbullying victimization is significantly associated with suicidal ideation among adolescents. Regarding demographic and health-related characteristics, all the variables included in the analysis were related to suicidal ideation. To the best of our knowledge, this is one of the few studies to use representative pooled data to examine the association between cyberbullying victimization and suicidal ideation among adolescents in Argentina, Panama, St Vincent, and the Grenadines. Our results have significant policy, and intervention implications for policymakers, educators, parents, and counsellors in schools and adolescents’ health and counseling services.

### Interpretation of the findings

Research on the psychological and mental health impacts of cyberbullying victimization and traditional bullying in general, especially among adolescents, continues to receive attention and interest significantly in recent times [10, 16, 19, 28]. Using the most recent data from the GSHS from three countries, namely Argentina, Panama, St. Vincent and the Grenadines, the results showed that 20% and 21% prevalence of cyberbullying victimization and suicidal ideation, respectively. The prevalence of cyberbullying victimization is higher than those reported in individual country studies in Vietnam (9%) [16], the US (4.8.–18.3%) [19, 33] and Hong Kong (11.9%) [28] but consistent with rates reported in another multi-country study in seven European countries (13.3–37.3%) [34]. The current study’s relatively higher cyberbullying victimization rate may reflect an increasing temporal trend among adolescents globally [10]. Importantly, in our analysis, suicidal ideation was somewhat higher, and the prevalence of suicidal ideation among victims of cyberbullying was higher than among non-victims. This finding is in line with a study conducted in Hong Kong, which reported that suicidal ideation was high among adolescents who were cyberbullying victims [28]. Studies and reports indicate that suicidal behaviors among adolescents globally have become a serious concern [10, 13]. Data from the 2017 Youth Risk Behavior Survey in the US suggest that 17.2% of adolescents seriously contemplated suicide, 13.6% planned how to attempt suicide, and 7.4% attempted suicide [14]. Elia et al. [15] reported suicidal thoughts and planning of 7.5% and 17.5%, respectively in 21 Latin American and Caribbean countries. Thus, it is essential to emphasize that the relatively high prevalence of cyberbullying victimization coupled with the significantly high likelihood of suicidality in cyberbullying victimization implies that more efforts toward prevention and intervention programs are needed.

Cyberbullying victimization has been demonstrated to be associated with adverse psychological and mental health, especially among adolescents [10, 16, 19, 28, 33, 34]. Our findings have shown that cyberbullying victimization has a significant association with suicidal ideation among adolescents after adjusting for potential confounders. Victims of cyberbullying in our sample were more likely to have suicidal ideation than non-victims. Our findings resonate with extant studies demonstrating that cyberbullying victimization is independently associated with suicidal ideation among adolescents. For example, using data from 1,963 students in 30 middle schools in the US, Hinduja and Patchin [19] found that cyberbullying victims were 1.9 times more likely to have attempted suicide. In another study among 4,886 Grades 7–12 Canadian students, cyberbullying victims were six times more likely to experience suicidal ideation [35]. Again, in representative cross-sectional data from a school-based sample of 3,522 Hong Kong adolescents, Chang et al. [28] found that cyberbullying victims were likelier to consider suicide seriously.

Though unsurprising, several plausible mechanisms may underline the association between cyberbullying victimization and suicidal ideation. Despite the positive correlation between cyberbullying victimization and suicidal behaviors, cyberbullying victimization does not directly cause suicide [19]. Some authors have argued that the incidence of health risk behaviors such as drug use, smoking, alcohol use, and lack of physical activity, among others, in adolescents needs to be closely examined in the association between cyberbullying victimization and suicidal ideation over time at the country level to make intervention policies more fitting and progressive [23]. Moreover, studies have demonstrated that many adolescents who experience suicidal ideation after experiencing cyberbullying victimization have other emotional, psychological, and social issues, such as academic struggle, low self-esteem, and depression [19]. Also, cyberbullying victimization tends to exacerbate instability and hopelessness in the minds of adolescents already struggling with stressful life circumstances [36]. Supportively, Bauman et al. [37], in their mediation analysis, found that depression partially or partly acts as a mechanism by which bullying affects suicidal ideation among students. All these explanations indicate the impact of additional factors that interact with cyberbullying victimization to cause suicidal ideation, and there are still more gaps that require further studies to identify and examine the contribution of factors such as stress, depression, and healthy lifestyles in the association of cyberbullying victimization with suicidal ideation. Future longitudinal studies should also seek to determine the role of conditioning factors that mitigate the association between cyberbullying victimization and suicidal risks. Though Chang et al. [28] performed a moderation and reported that life satisfaction partially mitigates the relationship between cyberbullying victimization and suicide ideation, their study was cross-sectional. In-depth qualitative studies are also needed to offer some lived experiences, explanations, and clarifications on the association between cyberbullying victimization with suicidal ideation.

Importantly, our findings show some significant associations between adolescents’ demographics, certain health-related factors, and suicidal ideation. Demographics such as age and gender and health behaviors, including cigarette smoking, drug use, tobacco use, loneliness, food insecurity, physical activity, peer support, and parental support, were significantly associated with suicidal ideation. Unsurprisingly, demographics such as gender and health behaviors such as loneliness, alcohol intake, tobacco use, drug use, cigarette smoking, and food insecurity associated with suicidal ideation. For instance, adolescents who were mostly lonely were seven times more likely to have suicidal ideation, which is consistent with earlier research [24]. Cyberbullying victimization is a serious experience that can lead to various psychopathologies, including feelings of loneliness [38]. Loneliness symptoms can impair one’s capacity to manage emotions and cause one to focus nearly entirely on negative parts of life [39]. The finding suggests that an approach to effective suicidal ideation prevention programs and policy should consider loneliness and other social isolations.

The fact that female adolescents are more likely to have suicidal ideation backs up previous research that revealed similar results that females have high rates rates of suicidal ideation [12]. It has been argued that females are more likely to use less dependable ways of suicide [40]. It has also been explained that though males may feel depressed and hopeless, they are less likely to admit and seriously consider suicide than females due to the perception that suicidal ideation is a sign of weakness and inadequacy in managing one’s affairs [41]. Several studies have also indicated that impulsivity, aggressiveness, anger, and hostility are crucial in explaining the gender differences in adolescents’ suicidal ideation because males are more likely to respond to frustration and stress with anger and hatred than females [42]. Our finding, therefore, implies that this relationship between gender and suicidal ideation cannot be overlooked. School-based suicidal ideation prevention programs and interventions should be developed and implemented through a gender lens.

A strong relationship between adolescents’ substance use and suicidal ideation was found in this study, which needs to be commented on. Adolescents who reported substance use such as cigarette, alcohol, and tobacco use were more likely to have suicidal ideation, which resonates with the growing evidence of substance use and suicide behaviors [43]. Empirical evidence suggests one main reason underlying the association between substance use and suicidal ideation. Substance use is likely to increase suicidal ideation risk because substance use may cause depressed feelings, decrease cognitive processing and problem-solving abilities, and influence adolescents’ essential associations and school/work performance [44]. The implication is that adolescents’ substance use is a severe public health issue and a significant predictive factor for suicidal ideation that needs to be addressed with evidence-based policies and interventions.

In line with other studies [45, 46], food insecurity is strongly associated with adolescent suicidal ideation. The mechanisms that connect food insecurity and suicidal ideation have been explained by a few hypotheses and theories [47]. Stuff et al. [46] have suggested that the potential stress and biological pathways can be responsible for the association between food insecurity and suicidal ideation. Feeling and experiencing food insecurity can be a source of embarrassment, anxiety, and stress [48], which may exacerbate mental disorders. In line with this argument, mental disorders among food inscured groups and individuals have been found [47]. Literature on dieting and starvation has hypothesized that malnutrition and micronutrient deficiency among food-insecured populations could explain the link between suicide ideation and food insecurity [49]. More research is needed to further explore the mechanism linking food insecurity and suicidal behaviors besides the commonly known risk factors, psychological factors, especially among adolescents.

Our findings also suggest the importance of physical activity on suicidal ideation among adolescents. Adolescents engaged in physical activity were significantly less likely to have suicidal ideation than those involved in physical activity. This finding is in line with other adolescent studies [50, 51]. A systematic review and meta-analysis of studies examining the relation between physical activity and suicidal ideation observed that being physically active is associated with lower suicidal ideation [52]. The association could be explained by the evidence that physical activity may protect suicidal-related risk behaviors by promoting positive emotional and psychological wellbeing [53], such as improvement in depressed mood, anxiety and stress, and self-esteem [54].

It has also been found that engaging in physical activity, especially among adolescents, promotes a positive self-image and protects against suicidality via its effect on psychological wellbeing [51]. Based on our findings and their consistency with previous results, we suggest that encouraging and promoting physical activity among adolescents may protect against their suicide-related risks. Interventions or studies for addressing suicidal ideation among adolescents should therefore consider physical activity.

Adolescents with parental and peer support and two or more friends had significantly lower odds of having suicidal ideation in our study, suggesting the importance of social networks and support regarding suicidal ideation among adolescents. Modern trends emphasize adolescents’ competence and need for independence. However, parental consent is critical in leading children to the next level of social functioning and promoting their mental health [16]. Previous research has found a link between peer support, parent-child relationships, and adolescent suicidal ideation, consistent with our findings [55]. Simultaneously, cross-sectional and longitudinal studies have found that increased peer support reduces suicide ideation with time [55]. The impact of parental and peer interactions on adolescent suicidal ideation should be factored into national programs to improve population mental health in this era of rapid technological development and changes. Similarly, organizing community activities that will enhance peer connectedness can assist in minimizing loneliness, anxiety, and suicidal ideation.

### Implications for prevention and intervention

Our findings inform/guide policymakers, parents/guardians, educators, school administrators, and clinicians working with adolescents and in adolescent health services. From our results, prevention strategies and intervention are required. As a part of prevention and intervention efforts, school authorities and policymakers should consider policies and measures that position adolescents beyond their school setting, as cyberbullying victimization and other lifestyle activities like alcohol use, tobacco use, and cigarette smoking may occur outside the community of care of their educational environment [37]. Moreover, our results call for gender-specific anti-cyberbullying initiatives, programs, and guidelines. These programs and initiatives could educate and counsel adolescents, especially females, on cyberbullying victimization and its effects. Education and awareness creation on effectively identifying and managing cyberbullying victimization should be provided to adolescents by educators and stakeholders such as parents, and counselors. In planning and implementing prevention and intervention for cyberbullying victimization, parents should be considered as parental support has proven to be a protective factor of suicidal ideation. Apart from prevention and intervention efforts, our findings suggest that there are unanswered questions regarding the underlying reasons for the association between cyberbullying victimization and suicidal ideation among adolescents that need further longitudinal and mixed method studies to enhance our understanding of this complex association, especially from the perspective of adolescents [56].

### Strength and limitation

The study’s main strength is that it is one of the first to report the relationship between cyberbullying victimization and suicidal ideation in Argentina, Panama, St Vincent, and the Grenadines in this specific age group. The study used representative data from a global school-based student health survey, improving the generalizability of its findings. The GSHS is a comprehensive and representative study of students’ health behaviors and risk factors. The study also used the most current data in these countries to ensure the findings reflect recent developments. However, our results should be viewed and interpreted in the context of some limitations associated with the study. The first limitation relates to the reliance on an adolescent self-report questionnaire, which subjects their responses to recall and social desirability bias. Second, our study did not evaluate any causal relationship between cyberbullying victimization and suicidal ideation because the GSHS data is cross-sectional and gathered at one time rather than multiple time points. Future studies should utilize longitudinal data collection methods to establish casual connections. In addition, our analysis excluded out-of-school adolescents and adolescents studying in private schools, thereby limiting the generalizability of the findings to all adolescents in Argentina, Panama, and St Vincent and the Grenadines. Finally, although using single items to represent the construct under investigation is not the best practice, such an approach has been widely demonstrated [37].

## Conclusion

cyberbullying victimization is significantly associated with adolescent suicidal ideation. This finding calls for developing and implementing evidence-based interventions and practices by school authorities and other relevant stakeholders to reduce cyberbullying victimization among adolescents. Protective factors such as parental support, peer support, and physical activity should be encouraged and enhanced among adolescents.

## Data Availability

The datasets generated and/or analysed during the current study are available in the https://www.cdc.gov/.

## List of abbreviations

GSHS: Global School-based Student Health Survey
WHO: World Health Organization
DHS: Demographic Health Survey
CDC: Centers for Disease Control and Prevention
US: United States
YRBS: Youth Risk Behaviour Survey
